# Vaping cessation support recommendations from adolescents who vape: a qualitative study

**DOI:** 10.1186/s12889-024-19036-1

**Published:** 2024-06-17

**Authors:** Lori Pbert, Catherine E. Dubé, Catherine S. Nagawa, Dante P. Simone, Jessica G. Wijesundara, Rajani S. Sadasivam

**Affiliations:** 1 Department of Population and Quantitative Health Sciences, UMass Chan Medical School, 55 Lake Avenue North, Worcester, MA 01605 USA; 2 Division of General Internal Medicine, Massachusetts General Hospital, Harvard Medical School, 100 Cambridge Street, Boston, MA 02114 USA

**Keywords:** Vaping, E-cigarette use, Adolescents, Reasons to quit, Cessation strategies, Qualitative study

## Abstract

**Background:**

Youth vaping is a serious public health concern, being more prevalent than any other tobacco use. To inform cessation interventions, we explored what adolescents perceive as their reasons for quitting and strategies to help them quit.

**Method:**

Semi-structured interviews were conducted with a convenience sample of 11 adolescents reporting vaping in the past 90 days and recruited from a high school in Massachusetts. Interviews were transcribed and dual-coded. Inductive thematic analysis was employed, and thematic summaries were prepared.

**Results:**

Reasons adolescents reported for quitting included cost, experiencing “nic-sick” from nicotine withdrawal or excess intake, negative impacts on mood, concentration, or health, and experiencing symptoms of nicotine dependence. Nearly all tried to quit multiple times. Barriers to quitting included exposure to vaping, access to vape products, stress, and “cool” new products or flavors. Quit strategies included avoiding others vaping, seeking social support to quit, addressing peer pressure to continue vaping, learning successful quit strategies from peers, and using distraction strategies or alternatives to vaping.

**Conclusion:**

Many adolescents who vape want to quit, and most have tried multiple times. Interventions need to engage adolescents with varying reasons to quit, barriers, and quit strategy preferences.

**Clinical trial registration:**

This study is registered through ClinicalTrials.gov. The trial registration number is NCT05140915. The trial registration date is 11/18/2021.

**Supplementary Information:**

The online version contains supplementary material available at 10.1186/s12889-024-19036-1.

## Background

E-cigarette use among U.S. adolescents is a serious public health concern, as noted by the FDA, with e-cigarette use exceeding combustible cigarette use [1]. The 2023 National Youth Tobacco Survey [2] found that 4.6% of middle school students and 10.0% of high school students reported current (past 30-day) e-cigarette use, with more than a quarter (25.2%) using e-cigarettes daily and over a third (34.7%) using them on 20 or more of the past 30 days, considered frequent use. Most adolescents (89.4%) use flavored e-cigarettes. Although e-cigarette use declined during the height of the COVID-19 pandemic, recent estimates suggest that the rates are again increasing [3] and e-cigarettes continue to remain the most commonly used tobacco product by youth [2].

Peer influence, as well as appealing flavors, have been identified as major factors in adolescent e-cigarette use. Adolescents do not recognize that most e-cigarettes contain nicotine [4], risking harm to brain development, impacting learning, memory, attention, mood, and impulse control, and increasing the risk of addiction [5]. Adolescents perceive e-cigarette use to be low risk, leading to a low intention to quit [6]. For those who have tried to quit, a national survey found that unassisted quitting was the most frequently reported method used [7]. In another study, few adolescents reported having used any cessation programs, and when they did, such as a text messaging program, they reported finding them helpful but withdrawing when they relapsed, highlighting the need to address lapses and increase engagement to improve treatment success [8].

Unfortunately, effective, evidence-based cessation interventions are lacking; to our knowledge, there is only one that exists, the This is Quitting texting program from the Truth Initiative [9–11]. While This is Quitting [11] has generated a high demand for cessation support and encouraging evidence of success, a variety of cessation tools are needed, especially for less motivated adolescents, including tools that focus on the pervasive influence of peers on e-cigarette use [6, 12]. This formative research was part of an NIH-funded pilot study developing peer-driven text messaging and coaching mobile intervention to support cessation for adolescents who vape (NIH 34 DA050992). We conducted in-depth qualitative interviews to explore adolescents’ reasons to quit vaping, prior attempts to quit, and their recommendations for cessation strategies.

## Methods

We detailed methods for this research in a previously published companion manuscript [13].

### Interview guide development

A qualitative, open-ended, semi-structured interview guide was developed. The interview guide was developed specifically for our study. Areas of inquiry included reasons to quit, advice to friends about quitting, prior quit experiences, and recommended strategies to help quit (Appendix 1).

### Setting and participant recruitment

A convenience sample was recruited from one public high school in Massachusetts from 513 students in grades 9 through 12, aged 13 to 19. High school enrollment comprised 82% White, 10% Hispanic, 5% multirace non-Hispanic, 2% African American, and 1% Asian, with a median household income of approximately $80,000. Flyers posted in the school, school-wide announcements, and materials mailed to parents were used. Parents could opt their child out if desired. Potentially eligible students who reported having vaped nicotine in the last 90 days were identified and screened. Assent (for those under 18 who were not opted out by their parents) and consent (for participants over 18 years old) procedures were conducted before enrollment and data collection. Participants were compensated with a $20 gift card.

### Data collection

Semi-structured open-ended interviews were scheduled for 1 h during the school day in a private room and audio recorded. A HIPAA-trained transcriptionist transcribed the audio recordings. All transcripts were reviewed and verified by a research team member.

### Analytic approach

Inductive thematic analysis was employed using a coding start list based on protocol questions (deductive coding) with additional coding driven by immersion in the qualitative data (inductive coding) [14]. Using Dedoose qualitative analysis software [15], two of the authors coded all interviews. Disputes in coding decisions were discussed until a consensus was reached. Coding tests for inter-rater reliability [16] resulted in a Kappa > 0.94. For each code (Table 1), reports, including all coded quotations, were generated, and code summaries were prepared by two team members, identifying areas of agreement and the range of responses for each code. Consensus code summaries formed the basis for the results reported below.

**Table 1:** Codes included in the analysis

Code Name	Definition
Reasons2Quit	Reasons participant or others should quit vaping. Includes: Benefits of quitting, what dislike about vaping, concerns about vaping (e.g., impact on important things in life like sports, ability to concentrate on schoolwork, financial costs, impact on mood, health, friendships, effect of nicotine)
Advice	Advice participant would give to family or friends thinking of starting or stopping vaping
PriorQuitAttempts	Reasons for deciding to try to cut back/stop, how often made attempts, what specific strategies were used, use of other tobacco product/vaping product to cut back/quit, strategies that helped/worked well when trying to cut back/stop vaping, supportive/helpful others, what made it hard to stop vaping (e.g., friends or family who interfered with efforts, mood/anxiety or stress), what made participant go back to using e-cigarettes last time tried to stop/vape less
QuitConfidence	What would help them become more confident in their ability to quit
QuitStrategies	If participant decided to quit, how would they go about it, what strategies do they think would help them stop
Technology-basedInterventions	Technologies that might help participant and friends quit vaping

## Results

### Participants

Thirty-two students were screened; 12 were eligible and enrolled. One participant dropped out, resulting in a final sample of 11. Three identified as female, 4 as male, 3 as non-binary, and 1 declined. The majority were 10th or 11th graders (*n* = 8), non-Hispanic White (*n* = 8), low income as defined by participation in the free lunch program (*n* = 7), had never smoked traditional cigarettes (*n* = 7), reported being addicted to vaping (*n* = 7), used flavors (*n* = 10), felt moderately to extremely confident in their ability to quit (*n* = 9), and smoked marijuana in the past month (*n* = 8). All vaped nicotine (*n* = 11). Half reported not thinking of quitting (*n* = 5), the others reported already being quit (*n* = 4) or considering quitting (*n* = 2).

### Qualitative results

Eleven individual interviews were conducted between March and June 2022 (average = 32.5 min; range 26–43 min). Five major themes related to adolescent vaping cessation were identified (Table 2 includes illustrative quotes in addition to those noted in the text).

**Table 2:** Selected themes, subthemes, and additional illustrative quotes

Themes	Subthemes	Illustrative Quotes
1 Reasons to quit	Cost	ID01 *They’re expensive…[ – you got to expend –] if you’re really addicted, you’re spending probably $100 a month on that just straight”* ID02 *It’s an expensive piece of electronics.* ID10 *It’s a big expense. You’re gonna do that and get addicted, then you’re going to be fresh outta cash.*
	“Nic-sick”	ID11 *Sometimes it’ll give you random headaches. Your stomach will randomly hurt. And obviously if you haven’t had it for a while, that doesn’t feel good.*
	Effects on physical health, including cancer risk	ID04 *I’ve heard it kind of makes your lung capacity… a little smaller. People say they have a harder time catching their breath.* *ID03 How bad it is for you… I wanted to go to the gym more, and I wanna be able to run longer distances without being outta breath.*
	Negative effect on mood and ability to focus	ID04 [When unable to vape] *I feel like it makes it harder to focus on things for sure. I feel like I become a little bit more irritable. People can get on my nerves a lot easier if I hadn’t had any nicotine … It can definitely make me feel annoyed, not so much angry as maybe frustrated… Anxiety a little bit.* ID01 *People get depressive mood swings and stuff like that because they don’t get nicotine*.
	Addiction	ID06 *I feel like my cravings have been just escalating, and I feel like when I get upset, I’m like, “Oh, my God, I need this.” I feel like I need it, in a way, and I feel like nicotine will help me, which – I know that it won’t, but in my head, I just think it will help me.* ID10 *My other friends definitely do have some problems with it [addiction]…’cause whenever we’re hanging out and there’s not vapes around, some of our friends will be like, “Oh, I wish we had it. I need it.”*
	Family and friend disapproval	ID06 *Kinda with family, ’cause they know I shouldn’t be doing it at school, and it kinda gets them upset.* ID07 *The stress and the anxiety and the fear that your parents found it.*
2 Advice to family or friends thinking of starting or stopping vaping	Advice to family or friends who want to start vaping	ID10 *First of all, why? Second of all, it’s a big expense. You’re gonna do that and get addicted, then you’re fresh outta cash.* ID11 *It’s really not worth it. You save a lotta money not doing it, save yourself a lotta trouble.*
	Advice to family or friends who want to stop vaping	ID02 *It’s gonna be hard, and you’re gonna wanna do it. Willpower and support is a lot of what you need. Honestly, I get that it’s an expensive piece of electronics, but throw it away.* ID09 *Probably to find candies or something small that you can hold on to…when you’re feeling the cravings. Find a game that you like to play.* ID05 *Just distract yourself a lot…pull yourself away from people that try and give you it.*
3 Prior experience with quitting vaping	Ever tried to vape less often or stop vaping and if so, number of times tried	ID12 *Two or three times….unsuccessfully.* ID11 *…failed four times.*
	What cutting back/stopping was like for them	ID01 *It was kinda hard, ’cause you have it around you all the time, people vaping around you.* ID11 *It’s terrible.* ID05 *It was pretty smooth. I smashed the device I had, and I was like, “I’m done.” And then I was done. That’s when I quit. I was just like, “All right, I’m done.”*
	Why decided to cut back/stop	ID01 *You know the health issues. Also, you just don’t feel like yourself sometimes when you’re vaping. It feels like that’s not you.* ID05 *Because I saw a video that a guy got put in the hospital, and I was like, “I don’t want that to be me.”*
	What helped quit	ID06 *Probably when I’m with my family, doing things as a family. We’re not thinking about any drugs or – we’re just having fun.* ID02 *Support from my friends, definitely, but mostly… willpower to not wanna be like my mother. I have a goal I need to upkeep.* ID05 *Friends, video games and candy helped.* ID03 *Not seeing other people do it or not having it be talked about.*
	What made quitting harder and reasons for relapsing	ID03 *Having all my friends do it.* ID05 *Everyone asking me if I wanted a hit.* ID12 *My family. They’re my main reason why I started. They’re very stressful, and once I started vaping…I could handle my family.* [I relapsed because] *I was really stressed out…and I was like, oh, OK. Never mind. I’m going back to this. I think it was actually a family thing, and my whole family was over, and I was all stressed out and stuff.*
4 Confidence in Quitting	What would help them become more confident in their ability to quit	ID12 *Seeing my friends do it [quitting] or having a group doing it with me, ’cause if my friends are all doing it and I’m the only one that’s not, it’s like, damn.* ID09 *Not being around it.* ID11 *Nothing, really. I’m confident. I just don’t want to.*
5 Strategies to Quit	Avoidance of others vaping and addressing peer pressure to not quit	ID05 *Pull yourself away from people that try and give you it.* ID12 *Throw away all my everythings of it and tell my friends that I’m going to quit and not to do it around me or talk about it.*
	Soliciting social support	ID03 *Yeah, talking with friends definitely, maybe a family member or…someone you’re close to.* ID12 *Having other people around you that’s going through the same thing is cool and also having one-on-one time if you need it.*
	Distraction strategies and alternatives to vaping	ID05 *Just distract yourself a lot.* ID01 *Try to find other means of stress relievers instead of vaping.* ID02 *I would wean myself off of it, and I would start chewing gum or going for a walk.* ID09 *…Look for other things to do like play games or draw or eat something… Stupid games or talking with friends, doing something that interests you.*
	Peer role models who had successfully quit	ID06 *I’ve had many friends that completely stopped and made it successful for them… the successful ones…they just said no to the people like, “No, I’m not doing that anymore.”*
	Use NRT or cannabis	ID12 *Chew gum. Nicotine patches if they can get them.* ID09 *I’ll probably look for other things to do like play games or draw or eat something or smoke weed.*
6 Technology-based Interventions	Technologies to help with quitting vaping	ID04 *Maybe a water-vapor thing, ’cause a lot of it is just the act of puffing on something*. ID01 *Any online things on social media.* ID09 *I probably would prefer things that didn’t have anything to do with it [vaping]. So, if we’re playing the video game, it would be nothing about vaping. … you would give scenarios for if you were doing the game, you could choose random scenarios that were semi-lighthearted or whatever.*

### Reasons to quit vaping

Many participants reported that the cost of e-cigarettes is a major reason for them to quit vaping. The students described vaping as expensive, pricey, a “waste of money” and “not worth it.” One student stated, *“It’s expensive…It’s really not worth it. You save a lotta money not doing it, save yourself a lotta trouble…”* (Participant 11). Another commented on the need to replace their vaping devices, noting *“It’s expensive…I don’t like that you can lose your vape a lot, because it’s small”* (Participant 09). And another student recognized the dual impact of vaping on both one’s health and finances, stating *“It’s not worth it to keep vaping. It can heavily affect your future like not only health-wise but money-wise”* (Participant 07).

Several participants noted that a potential reason for quitting is experiencing what they called “nic-sick”. Nic-sick was described as unpleasant physical symptoms, primarily nausea or headaches, attributed to nicotine withdrawal or excessive nicotine intake from vaping. “*Sometimes it makes me feel a little sick, so that can sometimes affect certain things like just hanging out with friends. They call it nic-sick. If I feel nic-sick, … I’ll kinda go quiet, ’cause I’m trying not to throw up, ’cause it makes you a little nauseous…I was chiefing…taking hit after hit…and I think I accidentally maybe took a couple too many hits, ‘cause I was in the car with my dad, and I started to feel sick. And I had to roll down the window and throw up”* (Participant 04).

Concerns about the effects of vaping on their physical health were also noted by the students as a reason to quit. This includes cough, reduced lung capacity and shortness of breath, asthma, sore or burned throat, as well as concerns about future health, including cancer risk. When asked about reasons for quitting vaping one student noted, *“Health concerns, lung cancer. You start getting really bad coughs. You might develop asthma due to the smoke…I realized that it was going to cause me future health problems”* (Participant 02). Another student reported being aware of the negative health effects of vaping, stating *“And it’s like I know it affects your health. Obviously, it’s not good for your lungs or anything like that. I know it can give you cancer and all that”* (Participant 04).

Many participants expressed significant concern about the negative impacts of vaping on their mood and ability to focus. This included irritability, frustration, “depressive mood swings,” worsening anxiety, and distraction or an inability to concentrate. Particularly disconcerting to students was the worsening of mental health symptoms, as well as the impact vaping had on their academic success, both of which were cited as significant reasons for quitting vaping. One student shared, *“I thought that vaping was helping my anxiety, but it really just made it worse…Sometimes it makes me more stressed…it’s like during the day I’ll just be thinking, ‘Oh, I wanna go home. I wanna go hit it’ and it would just completely get me off-track from my schoolwork.”* (Participant 07). Another noted, “I feel like it [vaping] gets in the way with school, and I feel like that’s a big problem with me” (Participant 06).

Addiction was seen as a significant drawback of vaping and a main reason to quit for many students. While some used the term “addicted” and “addiction,” many simply reported symptoms of nicotine dependence without associating their experience with dependence. It was described as an unwelcome feeling such as significant cravings to vape: “*I just thought it would be cool, and then I didn’t realize that it would get me addicted to it. [I dislike] how I feel like I need it. I feel desperate, in a way. I need it…and then when I actually do it [vape], then I’m like, is it really worth it?”* (Participant 06). Another student noted, *“I just kinda think about it a lot, and then once I finally do vape, I feel better. So [that’s] what addiction is.”* (Participant 12). Students noted cravings occur when they are unable to vape, see others vape, when stressed, and when they realize they do not have access to vaping products. When unable to vape, other symptoms included feeling desperate, anxious, depressed, impatient, restless, unable to concentrate, irritable, and headaches. A few participants reported never experiencing cravings to vape: *“I don’t think I really need to vape. I’ve never had the feeling that I needed to vape unless I was in a very bad mental spot. It’s not necessarily the need to vape. It’s just the need to feel something else.”* (Participant 09). Even when students report not experiencing addiction themselves, they recognize symptoms of dependence in their friends and describe it as an unappealing behavior: *“I don’t like that my friends who are really addicted to it, they get irritated when they don’t have it, or that’s all they’ll talk about”* (Participant 09).

Only a few participants discussed family disapproval as a significant negative aspect of vaping and reason for quitting. Anticipated family reactions ranged from being upset to *“If they ever found out, I would be chalked up on the side of the road”* (Participant 10). Only one student brought up friend disapproval or losing friends due to vaping as a drawback of vaping and as a reason for quitting: *“Specific people won’t respect you because of what you do [vaping]…I’ve had a couple of ‘em [friends]”* (Participant 10). Another felt that being viewed as a vaper by others was viewed as a negative by others and they did not want to be seen that way: *“I don’t like that I vape most of the time…I don’t want to be seen like that at school…at school, I prefer to be seeing in a more professional way, and I feel like that isn’t vaping”* (Participant 09).

### Advice to family or friends thinking of starting or stopping vaping

When asked what advice they would give to a family member or friend who expressed the desire to start vaping, most participants stated they would tell them not to start vaping, that starting to vape is not worth it for many reasons, including affecting your health, the cost associated with vaping, and that vaping leads to your getting addicted and relying on it. One participant stated, *“I’ll probably tell them it’s not worth getting addicted to, ‘cause you feel like you rely on it and you need it to survive, and it’s not a good feeling.”* (Participant 06). Another noted they would say, *“Don’t do it. It messes with you. It may seem cool, but it’s really not.”* (Participant 05). One participant noted they would not give friends or family advice as it is futile: *“I wouldn’t, ‘cause when – just like any other kind of drug, once you start, you can’t really stop. I mean, you can, but it’s gonna affect you forever.”* (Participant 12).

For a friend or family member who is interested in quitting vaping, several participants stated they would provide this person with encouragement and support to quit, including noting that it is going to be difficult and requires willpower. For example, *“I’d encourage them. I’d give them all my support. I can see why they wanna quit. They wanna do better.”* (Participant 06). Many shared that they would provide the person interested in quitting with specific strategies to quit, such as eating candies, playing games, avoiding being around vaping, distracting themselves, finding other ways to reduce their stress, and recognizing the costs associated with continuing to vape. For example, *“Try to stop staying around it. Try to find other means of stress relievers instead of vaping.”* (Participant 01). Using social support was also suggested: *“Stick to your friends for support and your family, too, ‘cause they’re the ones that – if you have friends that don’t do it, they’ll be very supportive of your stopping. But your family should definitely.”* (Participant 11). One participant said they would not give advice to someone else, noting it would sound preachy and superior: *“I would probably say not to, just because…in the long term, it’s probably not good for you. But who am I to judge? So, if somebody wants to do it, I don’t really care…I feel like a lot of people, when they try to help people quit, they’re kind of preachy about it…they kind of act like they’re coming from a place of superiority because they dont.”* (Participant 04).

### Prior experience with quitting vaping

All participants except two reported having tried many times (2–4 or more times) to vape less often or stop vaping altogether. For example, one student noted, *“I’ve tried many times, but I just give up, and I feel like that I just need it”* (Participant 06). Of those students who had tried to quit or cut back, the majority noted that it was very difficult: *“It was hard. I tried to only hit it three times at most three sessions, I guess, a day, and I found myself being really irritable and stressed and couldn’t really handle people”* (Participant 12). Only two stated they had not tried to quit, with one stating: *“No…because I don’t really do it that often as it stands. And quitting altogether, I could do that. I just don’t really feel or see the need to”* (Participant 10). Only one participant reported successfully quitting with no difficulty after one try.

When asked why they had decided to cut back or stop vaping, several students reported it was in response to concern about future health problems: “*Because I realized that it was going to cause me future health problems, and as one that does not like needles, I would rather not deal with needles”* (Participant 02). One participant noted trying to quit due to cost, and another feared being suspended from school: “*’Cause I got suspended for the first time, and I got scared”* (Participant 11).

What helped students in their efforts to quit vaping varied greatly across participants, with no one strategy standing out. Helpful strategies reported by students included being with and having fun with their family, being with their friends, willpower, finding ways to reduce their stress, avoiding others who were vaping or talking about vaping, and using marijuana. Having friends who were also trying to quit was particularly helpful: *“Honestly, friends telling me that they’re trying to quit, too, motivational”* (Participant 01). As stress was a significant reason for vaping, managing stress was an important quit strategy: *“Trying to cut out other stressors in your life, ‘cause stress is a big factor of why I started”* (Participant 12).

Students reported several things that made quitting more difficult for them and led to their relapsing. Challenges to quitting included being exposed to others who were vaping, thinking about quitting, family stress, stress from being suspended, and learning about new e-cigarette flavors. One student noted, *“It was kinda hard, ‘cause you have it around you all the time, people vaping around you”* (Participant 01). Being exposed to social media talking about new flavors was particularly difficult for some when trying to quit: *“…I would notice…’Oh, this sounds like a really good flavor. I wanna try that.’…so then I bought one, and I was like, OK, this is my last one….And then I opened social media, and I found this one that looked really cool. I’m like, OK, this is gonna be my last one. And it was not.”* (Participant 07). Not surprisingly, many of these challenges were also cited as reasons for resuming e-cigarettes the last time they tried to stop, including stress, family stress, seeing a new “cool” vaping product, and access to vape products through friends. One did not know why they resumed vaping. No common reasons for relapsing were identified, highlighting the need to assess reasons for relapse individually for each adolescent.

### Confidence in their ability to quit

When asked what would help them become more confident in their ability to quit, students mentioned that having an optimistic attitude, achieving goals that they set for themselves, and quitting with their friends would increase their confidence in their ability to quit vaping. Others indicated that avoiding those who vape would increase their confidence: *“Probably stay away from people that do it”* (Participant 03). A few participants reported that they already had sufficient confidence in their ability to quit but were not interested in quitting: *“I am so confident in my ability to quit. I could do it if I wanted to. I just don’t want to because I really just don’t care.”* (Participant 10).

### Strategies to quit

Many participants made concrete suggestions for strategies to quit, including eating candies, playing games, avoiding being around vaping, distracting yourself, finding other ways to reduce stress, as well as recognizing the cost of continuing to vape. The latter ties in with participants reporting that cost was a major reason for quitting vaping.

Avoiding being around people who are vaping and places where vaping is occurring, along with not asking friends for a vape, were key strategies noted. One participant shared, *“Try to stop staying around it”* (Participant 01). Another stated, *“Probably not going to the bathroom at all, ‘cause that’s where everyone does it”* (Participant 06). This student also noted the importance of addressing peer pressure to not quit, which can be a significant barrier to successfully quitting vaping: *“Some friends will try to push it like, ‘Are you sure? Are you sure?’ and…if I’m really focused on trying to quit, I’ll be like, ‘No. I said no”* (Participant 06).

Many participants reported getting support from their social network, including friends or family members, to help them quit and having others quitting with them would be particularly helpful: *“Stick to your friends for support and your family, too, ’cause they’re the ones that – if you have friends that don’t do it, they’ll be very supportive of you stopping. But your family should definitely’* (Participant 11).

Finding ways to distract themselves from cravings and things to do instead of vaping was frequently mentioned. This included things like playing games, chewing gum, eating a mint, going for a ride, and doing something of interest. One participant noted, *“I try to not think about it. So, I try to find things to distract me like cleaning my room or doing homework”* (Participant 06). Another suggested, *“Chew gum. Do other things, hobbies that make you happy”* (Participant 12).

A few participants noted that learning of quit strategies from peers who had successfully quit was a helpful strategy, seeing them as peer role models. They mentioned not knowing much about the process of quitting and related challenges and barriers, so having others share their quitting journey was important. One student noted, *“I’ve had many friends that completely stopped and made it successful for them”* (Participant 06).

Many participants reported having adults in their lives who had used nicotine replacement therapy (NRT) to quit smoking cigarettes. For example, “*My aunt uses nicotine gum. She has used it for a while. My friends probably don’t even know that exists”* (Participant 10). They assumed that NRT could be used by adolescents in quitting vaping, despite NRT not being FDA-approved for those under 18 years of age in the U.S. A few suggested using cannabis to help with quitting, reporting a similar calming effect to vaping nicotine: “*[CBD] gives a similar calming effect without having the negative side-effects of breathing in things”* (Participant 11).

When asked about technology-based interventions or approaches that might help them and their friends quit vaping, most participants endorsed text messaging. The kinds of text messages that participants recommended included providing tips to help them not to vape. For example, *“If somebody’s trying to stop vaping, then you could give them tips on how to stop and then slowly move on to, ‘What do you do if you’re craving it?’. Or if they’re in the process already of trying to quit, then it’s just, ‘If you need advice, then…instead of nicotine, here’s something that you can do’”* (Participant 07). The participants did not mention including the use of risk messages in the texts. Instead, they mentioned using text messages to send positive, motivational messages. One participant noted, *“Positive words…You don’t wanna use negative words so that they feel in a negative mood about it. You wanna say, ‘Hey, you can get through this’ type of thing.”* (Participant 01). Another suggested asking about their cravings and whether they had vaped: *“Maybe asking what the crave rating is on the day like 1 through 10, how many times you’ve had – wanting to smoke or something…if you have smoked that day, if you havent”* (Participant 12). Other suggestions included the use of social media, email, pop-up ads, a timer that regulates vaping intake, a placebo vaping device that is only water, and a video game that has nothing to do with vaping, intended as a distraction. For example, one student suggested, *“Email, because everyone’s refreshing their emails a lot. Yeah, that’s probably about it, text messages, videos.”* (Participant 05).

## Discussion

Most of the adolescents interviewed reported wanting to quit vaping and having tried unsuccessfully to quit many times. This finding is consistent with prior studies, which have found that more than half of adolescent e-cigarette users report at least one past serious quit attempt [17, 18] and there is high interest in quitting e-cigarette use among adolescents [17, 19], suggesting an unmet need for vaping cessation interventions.

Common reasons to quit vaping reported included cost, cravings, and symptoms of nicotine addiction (getting “nic-sick” from excess nicotine intake or withdrawal). Concern about cost and nicotine dependence as reasons for quitting vaping is consistent with other studies [20, 21]. Other common reasons to quit cited by our study participants included significant concerns regarding the effect of vaping on their health and their mood, including worsening of anxiety, depressive symptoms, stress, and their ability to concentrate. Of note, many reported using vaping to try to reduce their symptoms of anxiety and stress. Anxiety disorders are the most common psychiatric condition in youth, affecting nearly 1 in every 4 adolescents [22]. Given that nicotine is known to produce an anxiolytic effect [23], it makes sense that adolescents would try vaping nicotine to manage their anxiety. Unfortunately, these adolescents also reported that vaping made their anxiety worse, not better, due to withdrawal symptoms experienced between vaping sessions.

Adolescents also noted many barriers to their being able to quit. One key barrier was that their friends vape, resulting in their being exposed to vaping and vaping products regularly. This is consistent with prior research, which found social influences to be a top trigger for use and a significant barrier to cessation [20, 24], and that peer use predicts both initiation as well as the continuation of adolescent vaping [25, 26]. Other barriers noted by our study participants included vaping for mood management, which is consistent with other research [24], and the appeal of new vaping products and flavors.

Adolescents shared many potential strategies to quit, including avoiding exposure to vaping, seeking support, learning from peers who have quit, resisting peer pressure to vape, and various distraction strategies. Interestingly, while some of these strategies were unique to our study participants, others have also been reported by youth and young adults who had successfully quit vaping, including the use of support systems and alternative coping mechanisms. For example, in one trial [24], the use of cold turkey, self-restriction, and alternative coping mechanisms were found to be the most frequently cited cessation strategies, and the use of support systems, apps, and education were found to be the most useful recommendations. Others also noted soliciting social support, friend or parental, as being a top cessation method [18]. In a national online study of adolescents who were current or former e-cigarette users [7], unassisted quitting was the most reported method used, with the next most frequently reported method being following advice from a friend. The latter is consistent with our finding that adolescents are interested in learning how to quit from their peers who have successfully stopped e-cigarette use. Encouragingly, that study also found that adolescents were open to using a variety of quitting methods.

In terms of technology-based interventions, we found that most participants endorsed text messaging. This is consistent with findings from the 2021 National Youth Tobacco Survey (NYTS), which found that most adolescents who made a quit attempt in the past year did not use any cessation resources but, of those who did, use of text messaging or a mobile app were one of the top cessation methods used [18]. The only texting program we are aware of that has shown encouraging evidence of success in helping youth quit is the This is Quitting texting program from the Truth Initiative [11]. However, those who elect to use this program are likely motivated youth. Cessation support tools are also needed to help those who are less motivated to quit, which is likely the majority of youth given adolescents perceive e-cigarette use to be low risk, leading to a low intention to quit [6]. Other technology-based interventions that may be worth considering are cessation interventions using social media, given the integral role social media plays in many adolescents’ lives. Indeed, our participants recommended using social media as a technology-based cessation tool. However, to date, there is limited evidence on the use of social media interventions to assist adolescents in their efforts to quit vaping [27].

Two strategies mentioned by our participants are not appropriate for adolescents, including nicotine replacement therapy, which is not FDA-approved for use in youth under the age of 18 and has not yet been found to be effective in helping youth quit, and the use of cannabis which is illegal in most states and all U.S. states under the age of 21. Some adolescents did discuss the dual use of e-cigarettes and cannabis, which needs to be systematically evaluated, as cannabis use potentially increases quitting difficulty and can lead to more serious psychoactive drug use [28].

There were several limitations to our study. First, recruitment was difficult due to challenges faced by schools during the COVID-19 pandemic. However, we were able to recruit 11 participants and reached saturation on many of the constructs assessed. Admittedly, our sample size was small, thus making evaluation of thematic saturation challenging. Thematic saturation was assessed by reviewing interview transcripts and checking for novel themes and concepts. Stories of e-cigarette cessation and preferences for intervention approaches were remarkably consistent across interviews. We concluded that saturation was achieved on many of the constructs assessed, including reasons for quitting and strategies to quit based on these reviews.

Second, there were few participants who had successfully quit vaping and maintained abstinence. Expanding the sample to include both successful quitters and those who struggle to quit in future studies will provide rich data on both challenges and efficacious strategies used by this population.

## Conclusion

E-cigarette use among U.S. adolescents is a significant public health concern. Most adolescents who vape want to quit and have tried multiple times but are impeded in their efforts due to significant social and emotional barriers, nicotine addiction, and the lack of accessible, evidence-based interventions. Adolescents expressed a range of reasons for wanting to quit, including the cost of vaping, experiencing what they call “nic-sick” from excessive intake and withdrawal of nicotine, the negative impacts of vaping on their mood, concentration, and future health, as well as the experience of nicotine dependence symptoms, which some adolescents recognize as addiction. Key barriers to quitting included having others around them vaping, easy access to vape products, stress, and the lure of enticing new products and flavors. In terms of strategies for quitting, avoidance of people who vaped and places where vaping occurred were key strategies noted by adolescents. Other strategies they recommended were to seek social support, the need to address peer pressure from some friends to continue to vape, finding ways to distract themselves from cravings, using alternatives to vaping, and learning quit strategies from peers who had successfully quit. Adolescents recommended using technology to support their efforts to quit, including text messages, social media, and email, and using video games for distraction. Implications of our findings include the need to design interventions that are accessible and engaging to adolescents that take into consideration their unique and varying reasons for quitting, barriers to stopping and remaining quit, and quit strategy preferences. The findings of this study provide insights to help healthcare professionals, public health and health promotion advocates, and other researchers in designing cessation interventions and tobacco control policies to help adolescents in their efforts to quit vaping.

### Electronic supplementary material

Below is the link to the electronic supplementary material.

Supplementary Material 1

## Data Availability

The data underlying this article cannot be shared publicly due to the concerns for the privacy of individuals who participated in the study. Deidentified data will be shared on reasonable request to the corresponding author and approval of the UMass Chan Medical School IRB. Proposals should be submitted to Rajani.Sadasivam@umassmed.edu.

## Appendix 1: Semi-structured interview guide

**Table:**

Construct	Questions
1. Reasons to quit (Reasons2Quit)	1.1 What do you dislike about vaping? 1.2 What are the DOWNSIDES of vaping? a. Makes you FEEL? b. Affects your MOOD? STRESS? c. Affects your SOCIAL LIFE? FRIENDSHIPS? DATING/RELATIONSHIPS? d. Affects your IMAGE - how you see yourself (SELF-IMAGE)? e. Affects other things important to you (sports, school, working out, other things you like to do)? f. Affects your HEALTH g. Affects your ability to CONCENTRATE on your schoolwork h. Other: cost, trouble at school or home, legal troubles, etc. i. Effect of nicotine 1.3 When you are unable to vape, what is that like for you? 1.4 When you cannot vape: 1.4.a. How do you feel? 1.4.b. Have you noticed other effects on: - Concentration - Irritability - Nervousness, restlessness - Anxiety
2. Advice to family or friends thinking of starting or stopping vaping (Advice)	2.1 What advice would you give family or friends who want to start vaping? 2.2 What advice would you give family or friends who want to stop vaping?
3. Prior quit attempts (PriorQuitAttempts)	3.1 Have you ever tried to vape less often or stopped vaping altogether? *[IF YES, HAVE TRIED TO VAPE LESS OFTEN/STOP ALTOGETHER]*: 3.1.a What was that like for you? a. Why did you decide to try to cut back or stop vaping? a. How many times have you tried to cut back or stop? b. What helped? c. What made it harder? d. What made you go back to using e-cigarettes last time you tried to stop/vape less? *[IF NO, HAVE NOT TRIED TO VAPE LESS OFTEN/STOP ALTOGETHER*]: 3.2.b Why not? – Can you tell me about others who have tried and what their experience was like? − Probe specific strategies including limiting amount of e-cigarettes use, avoiding others who vape, setting a quit date and plan, speaking with health professional or trusted adult, using nicotine gum, a nicotine patch, or other quit-aid medications 3.3 What do you think makes it easier to cut back or stop vaping?
4. Confidence in quitting (QuitConfidence)	4.1. What would help you become more confident in your ability to quit?
5. Strategies to quit (QuitStrategies)	5.1 If you were to decide to try to stop vaping, how would you go about it? If tried to quit in the past: 5.1.a What helped? If did not try to quit in the past, others’ experience: 5.1.b Can you tell me about others who have tried and what their experience was like? − Probe specific strategies including limiting amount of e-cigarettes use, avoiding others who vape, setting a quit date and plan, speaking with health professional or trusted adult, using nicotine gum, a nicotine patch, or other quit-aid medications
6. Technology-based interventions that might work: Texting, Videos, PeerCoach, OtherTech	6.1 What *other types of technologies* or approaches might work well for you and your friends who vape? [other than the texting program and videos proposed]
