# Supplementary material for: Vaping cessation support recommendations from adolescents who vape: a qualitative study
Source: BMC Public Health. 2024 Jun 17;24:1615. doi: 10.1186/s12889-024-19036-1 (PMC11181636; doi:10.1186/s12889-024-19036-1)
Supplement: Supplementary file 1 — Supplementary Material 1 [file 12889_2024_19036_MOESM1_ESM.docx]

**APPENDICES**

**Appendix 1. Vaper-to-Vaper Interview Guide**

**Participant ID#:**

**Date/Time:**

**Interviewer:**

**Format:** Semi-structured individual interviews

**Purpose:**  To elicit the adolescent’s experience with vaping (e.g., concerns, motivations, nicotine dependence, attempts to quit/cut back) *[and to facilitate their input into the Vaper-to-Vaper intervention development to inform design and adaptation of the toolset – Results reported separately]*

Interviews may be conducted in-person, by phone via Zoom, or using online video via Zoom.

**Materials: In-Person**

- Recorders (2) – charged/backup batteries
- Mics (2) – backup batteries
- Materials packet for facilitator
- Sign and tape for Door – “Interview in Progress”
- Observer Notes Form [if needed]
- Gift card
- Participant packet (pre-marked with participant ID) – Assent Fact Sheet, survey (if not completed in advance)
  **Materials: Phone or On-Line Video**
- Phone/video recorder (2) – charged/backup batteries
- Materials packet for facilitator
- Sign – “Interview in Progress”
- Observer Notes Form [if needed]
- Mail/email participant packet to participant (pre-marked with participant ID) – Fact Sheet, survey (if not completed online)
- Link to online gift card

*[INSTRUCTION TO INTERVIEWER:* *Prior to the interview, privately review survey and ensure accurately completed - e.g., age incorrectly entered. Also, identify any areas you wish to clarify when get to that section of the interview - e.g., high vaping frequency].*

INTERVIEWER INTRO:

Thank you for taking the time to meet with me today. And thank you for agreeing to be a part of our Advisory Panel for the research project.

This interview will last about an hour. We will learn from this interview and others by listening to the thoughts and ideas you talk about. We will look for overall themes or topic areas that emerge over all the different interviews that we are conducting. We will look to see if there is agreement between people, or if there are a wide range of ideas or positions. Your responses will be shared outside of our research group only in summary along with the other interviews and no one will ever know that you said a particular comment. To ensure your confidentiality, I’d like to refer to you by your first name only – or any nickname or other name you might prefer. *[Clarify preferred name].* You can call me *[first name]*.

I am going to record this discussion to help me capture all of your comments. We will transcribe the recording and use the typed transcript to compare what all the participants have said and to prepare our report. Once we have completed using the typed transcripts, we will delete this audio recording.

**Begin Recording and Review Fact Sheet**

Are you ready to get started? If so, let’s begin. I am now turning on the recorders. ** TURN RECORDERS ON **

*[State the date and time on the recording]* I would like to begin by going over the assent form to be sure you still want to participate*. [Review assent form].* I want to emphasize that your participation is voluntary, that you can decide not to participate with no penalty, and that you can withdraw at any time. You can skip any questions that you do not want to answer. I also want to mention that we will not share any information you might give us about illicit drug use with law enforcement agencies, your parents, the school, or others. We will, however, have to let someone (like the school nurse) know if you plan to harm yourself or others. Any questions? *[STOP if participant withdraws consent – provide gift card].*

**Start Interview**

**I wanted to start by asking you how you talk about your vaping or e-cigarette use. What do you call it?** *[Use participant’s preferred terms throughout – replace the word VAPING in brackets below].*

So, what I’d like to hear from you are your own thoughts, opinions, and experiences. You are the expert here – and there are no wrong answers! What I’d like to learn about today is your experience starting [vaping] – when and if you realized [vaping] had become a habit for you, and what that was like – and any thoughts you have about how high school students like you might try to cut back or quit [vaping].

| **Construct** | **Questions** |
| --- | --- |
| 1.Reasons to quit (Reasons2Quit) | 1.1 What do you dislike about vaping?  1.2 What are the DOWNSIDES of vaping?   1. Makes you FEEL? 2. Affects your MOOD? STRESS? 3. Affects your SOCIAL LIFE? FRIENDSHIPS? DATING/RELATIONSHIPS? 4. Affects your IMAGE - how you see yourself (SELF-IMAGE)? 5. Affects other things important to you (sports, school, working out, other things you like to do)? 6. Affects your HEALTH 7. Affects your ability to CONCENTRATE on your schoolwork 8. Other: cost, trouble at school or home, legal troubles, etc. 9. Effect of nicotine   1.3 When you are unable to vape, what is that like for you?  1.4 When you cannot vape:  1.4.a. How do you feel?  1.4.b. Have you noticed other effects on:  - Concentration  - Irritability  - Nervousness, restlessness  - Anxiety |
| 2. Advice to family or friends thinking of starting or stopping vaping (Advice) | 2.1 What advice would you give family or friends who want to start vaping?  2.2 What advice would you give family or friends who want to stop vaping? |
| 3. Prior quit attempts (PriorQuitAttempts) | 3.1 Have you ever tried to vape less often or stopped vaping altogether?  *[IF YES, HAVE TRIED TO VAPE LESS OFTEN/STOP ALTOGETHER]:*  3.1.a What was that like for you?   1. Why did you decide to try to cut back or stop vaping? 2. How many times have you tried to cut back or stop? 3. What helped? 4. What made it harder? 5. What made you go back to using e-cigarettes last time you tried to stop/vape less?   *[IF NO, HAVE NOT TRIED TO VAPE LESS OFTEN/STOP ALTOGETHER*]:  3.2.b Why not? – Can you tell me about others who have tried and what their experience was like?   - Probe specific strategies including limiting amount of e-cigarettes use, avoiding others who vape, setting a quit date and plan, speaking with health professional or trusted adult, using nicotine gum, a nicotine patch, or other quit-aid medications   3.3 What do you think makes it easier to cut back or stop vaping? |
| 4. Confidence in quitting (QuitConfidence) | 4.1. What would help you become more confident in your ability to quit? |
| 5. Strategies to quit (QuitStrategies) | 5.1 If you were to decide to try to stop vaping, how would you go about it?  If tried to quit in the past:  5.1.a What helped?  If did not try to quit in the past, others’ experience:  5.1.b Can you tell me about others who have tried and what their experience was like?   - Probe specific strategies including limiting amount of e-cigarettes use, avoiding others who vape, setting a quit date and plan, speaking with health professional or trusted adult, using nicotine gum, a nicotine patch, or other quit-aid medications |
| 6. Technology-based interventions that might work: Texting, Videos, PeerCoach, OtherTech | 6.1 What *other types of technologies* or approaches might work well for you and your friends who vape? [other than the texting program and videos proposed] |

**Closing the Interview**

We have reached the end of our time together. But before we stop:
- Did I miss anything?
- Anything else you would like to tell me -- about vaping, or anything else we’ve been talking about?
Great, thank you. It sounds like we are done… I am now going to turn off the recorders.

** TURN OFF RECORDERS **

I want to thank you for participating in this interview. I have learned a lot from you! As I mentioned before, this interview will be transcribed and then analyzed with all our other transcripts. Once we have the transcription, we will remove your name and replace it with a participant number to further protect your confidentiality.

Thank you again for a great discussion. (Sometimes people mention new things after the interview is over. Be sure to keep notes documenting these additional comments and let the participant know you are collecting them. “This is great stuff – if it is OK I am going to write these ideas down and add this to your transcript. Is that OK with you?” – another option is to keep them anonymous if need be.)

*[Instruction to the Interviewer]: If the participant makes relevant spontaneous comments after the recorders are stopped, ask, “Is it OK if I write that down?” and make a written record of those comments.*

***Provide gift card*
